# Femoral overgrowth in children with congenital pseudarthrosis of the Tibia

**DOI:** 10.1186/s12891-016-1157-x

**Published:** 2016-07-12

**Authors:** Mi Hyun Song, Moon Seok Park, Won Joon Yoo, Tae-Joon Cho, In Ho Choi

**Affiliations:** Department of Orthopedic Surgery, Jeju National University Hospital, 15 Aran 13-gil, Jeju-si, Jeju South Korea; Department of Orthopedic Surgery, Seoul National University Bundang Hospital, 82 Gumi-ro, 173 Beon-gil, Bundang-gu, Seongnam-si, Gyeonggi-do South Korea; Division of Pediatric Orthopaedics, Seoul National University Children’s Hospital, 101 Daehak-ro, Jongno-gu Seoul, South Korea

**Keywords:** Congenital pseudarthrosis of the tibia, Femoral overgrowth, Distraction osteogenesis, Neurofibromatosis

## Abstract

**Background:**

Having observed a tendency towards femoral overgrowth (FO) of the affected limb in children with atrophic-type congenital pseudarthrosis of the tibia (CPT), we aimed to identify the incidence of, contributors to, and patterns of FO among such children.

**Methods:**

We retrospectively evaluated 55 children with CPT, 22 with prepseudarthrosis and 33 with atrophic-type CPT from 1989 to 2012. FO was defined as an affected femoral segment ≥10 mm longer than the contralateral segment. We investigated FO incidences in prepseudarthrosis versus atrophic-type CPT. Sex, laterality, coexistence of neurofibromatosis type 1, development of frank pseudarthrosis, extent of tibial shortening, shortening in foot height, deformity severity, distraction osteogenesis (DO) treatment, refracture, increased femoral neck-shaft angle, tibiofemoral angle, and ankle valgus angle were investigated to identify potential contributors to FO. Patterns of FO were also determined.

**Results:**

At initial presentation, 11 patients exhibited a mean of 13 mm (10–23) of FO. However, the nature of FO changed over time during the follow-up period (mean, 10.8 years; range, 4.3–19.3). At the last follow-up, 14 patients presented with a mean of 12 mm (10–18) of FO. With the exception of one patient, all patients with FO presented with atrophic-type CPT. Frank pseudarthrosis, DO treatment, and increased femoral neck-shaft angle were significantly associated with FO (*p* = 0.016, *p* = 0.001, and *p* = 0.005, respectively). Diverse patterns of FO were observed.

**Conclusions:**

FO of the affected limb is frequently encountered in patients with atrophic-type CPT. A compensatory response to frank pseudarthrosis, DO treatment, and neurofibromatosis may play a role in the diverse patterns of FO.

## Background

The vast majority of patients with congenital pseudarthrosis of the tibia (CPT) present with a dysplastic tibia with an anterolateral bow at birth [1, 2]. In a few cases, this condition has a benign course of nondysplastic anterolateral bowing; however, most individuals with this condition undergo dysplastic changes, including failure of tubulation and a widened medullary canal, or cystic prefracture or canal enlargement from a prior fracture [3, 4]. This prefracture status is called ‘prepseudarthrosis’. Once a fracture occurs, there is little tendency for the lesion to heal spontaneously, and frank pseudarthrosis with atrophic ends, the so-called ‘atrophic-type CPT’, usually results [2]. Therefore, it may be more appropriate to describe this entire phenomenon as anterolateral bowing of the tibia with congenital dysplasia rather than CPT [1–3, 5] because it is obviously a heterogeneous entity with different prognoses [1].

The treatment of atrophic-type CPT is challenging. Despite marked improvement in the primary healing rate as a result of modern treatment methods, the residual problems are often perplexing and demanding [2, 6, 7]. We have used the Ilizarov technique for atrophic-type CPT since the late 1980s. This technique enables a multi-targeted approach to osteosynthesis, axial realignment, ankle mortise stabilization, and limb length equalization. Interestingly, we have observed a tendency toward femoral overgrowth (FO) of the affected limb in atrophic-type CPT compared with prepseudarthrosis. In the literature, only two previous reports briefly addressed FO of the affected limb in CPT, especially in atrophic-type CPT [5, 8]. It is the intention of this study to investigate the incidence of FO in patients with atrophic-type CPT versus prepseudarthrosis. We hypothesized that patients with atrophic-type CPT would have a higher incidence of FO compared with those with prepseudarthrosis. We also attempted to identify potential contributors to FO and to determine various patterns of FO.

## Methods

This study is a retrospective review of children with CPT who were treated at our institution from 1989 to 2012. Patients diagnosed with CPT and followed until preadolescence or adolescence were included; patients who were at least 10 years of age at initial assessment, had incomplete medical records and radiographs with a relatively short follow-up, and/or had generalized hemihypertrophy of the lower limb caused by neurofibromatosis were excluded. A total of 55 CPT patients (26 boys and 29 girls) who were unilaterally affected (on the right and left sides for 24 and 31 patients, respectively) were enrolled in this study. Forty-nine patients began their treatment at our institution, and the remaining six were referred to us after the failure of previous osteosynthesis attempts. At initial presentation, 16 patients were classified as Crawford Type I; 6 as Crawford Type II; 3 as Crawford Type III; and 30 as Crawford Type IV [9]. Thirty-nine patients had neurofibromatosis type 1 (NF1); the remaining sixteen did not. The mean age at initial presentation was 2.8 years (0.1–7.3), and the patients were followed for 10.8 years (4.3–19.3). Twenty-two patients remained in the prepseudarthrotic stage during the follow-up period; in contrast, 33 patients exhibited atrophic-type CPT. Table 1 summarizes the characteristics of the patients with prepseudarthrosis versus atrophic-type CPT.

**Table 1:** Patient characteristics of prepseudarthrosis versus atrophic-type CPT

Variables		Prepseudarthrosis	Atrophic-type CPT	
		(*n* = 22)	(*n* = 33)	*p*-value
Age at initial presentation (years)		3.0 (0.1–4.5)	2.6 (0.1–7.3)	0.595^a^
Sex (Male: Female)		13 : 9	13 : 20	0.152^b^
Laterality (Right: Left)		9 : 13	15 : 18	0.739^b^
Neurofibromatosis type 1 (Presence: Absence)		10 : 12	29 : 4	0.001^b^
Crawford classification (I: II: III: IV)		16 : 6 : 0 : 0	0 : 0 : 3 : 30	<0.001^b^
Femoral overgrowth	At initial presentation	1 : 21	10 : 23	0.019^b^
(With: Without)	At last follow-up	1 : 21	13 : 20	0.004^b^

*CPT* congenital pseudarthrosis of the tibia

^a^Independent t-test, ^b^Fisher’s exact test

Among the 22 patients with prepseudarthrosis, two patients underwent prophylactic bypass grafting, 10 patients wore an ankle-foot orthosis or a short leg cast, and the remaining 10 patients were simply observed without any treatment. In contrast, all 33 of the patients with atrophic-type CPT underwent the authors’ fibular status-based Ilizarov treatment [6] for osteosynthesis alone (*n* = 9) or for osteosynthesis in addition to distraction osteogenesis (DO) of the tibia/fibula (*n* = 24). Twenty-four patients underwent 31 rounds of DO of the affected tibia, with three rounds of DO in 2 patients, two rounds of DO in 3, and one round of DO in 19. The mean age at surgery was 4.5 years (1.7–7.3).

FO was defined as an affected femoral segment ≥10 mm longer than the contralateral segment based on a slit scanogram (as described by Bell and Thompson) [10], whereas a femoral segment length discrepancy of <10 mm was regarded as non-significant [5]. The incidences of FO in the prepseudarthrosis group and the atrophic-type CPT group were compared. The nature of the FO was classified as either FO that was consistent during follow-up (Type A); FO that was not observed at the initial presentation but that developed and remained during follow-up (Type B); FO that was observed at the initial presentation but that was not apparent during follow-up (Type C); and FO that developed after the initial presentation and subsequently resolved (Type D).

To identify potential contributors associated with FO, we investigated the following variables: sex, laterality, coexistence of NF1, development of frank pseudarthrosis, extent of tibial shortening, shortening in foot height, severity of the deformity, DO treatment, refracture after Ilizarov osteosynthesis, increased femoral neck-shaft angle, tibiofemoral angle, and ankle valgus angle. Tibial length of the affected limb was replaced by effective tibial length [11], which was measured from the medial plateau to the plafond; the extent of tibial shortening was then measured as the difference between the tibial lengths of the unaffected and the affected limbs. Shortening in foot height was defined as an affected foot height ≥5 mm shorter than the contralateral foot height; foot height was measured from the talar dome to the floor [12]. Deformity severity was determined based on the length of the atrophic segment, including the pseudarthrosis portion, as a percentage of the entire length of the affected tibia. The femoral neck-shaft angle was defined as the angle between the axis of the femoral neck passing through the center of the femoral head and the axis of the femoral shaft [13], and an increased femoral neck-shaft angle was defined as an increase of ≥10° in the femoral neck-shaft angle of the affected limb compared with the contralateral limb. The tibiofemoral angle was defined as the angle formed by the axis of the femur and the axis of the tibia [14], whereas the ankle valgus angle was regarded as the angle between the axis of the tibial shaft and the tibial plafond line [15]. We also examined the effect of an increased femoral neck-shaft angle on FO. This length-gain effect was calculated by subtracting the distance between the summit of the femoral head and the mid-level of the lesser trochanter of the unaffected limb from that of the affected limb.

We assessed patterns of FO to determine whether femoral segment length discrepancies for the affected limb followed any of the developmental patterns of limb length discrepancy that were previously reported by Shapiro [16].

### Statistics

We used the independent t-test for continuous variables and Fisher’s exact test for categorical variables to compare the characteristics of patients with prepseudarthrosis with those of patients with atrophic-type CPT. To identify potential contributors associated with FO, we analyzed numerical variables using independent t-test, whereas categorical variables were analyzed using Fisher’s exact test. We then performed multivariate logistic regression analysis with FO as the outcome variable; dependent variables were those with *p*-values <0.05 on the univariate analysis or those with clinical significance. Statistical analyses were performed using SPSS, ver. 21.0 (SPSS, IBM Corp., Chicago, IL); *p*-values < 0.05 were regarded as statistically significant.

## Results

### Incidence of femoral overgrowth

**Fig. 1:**
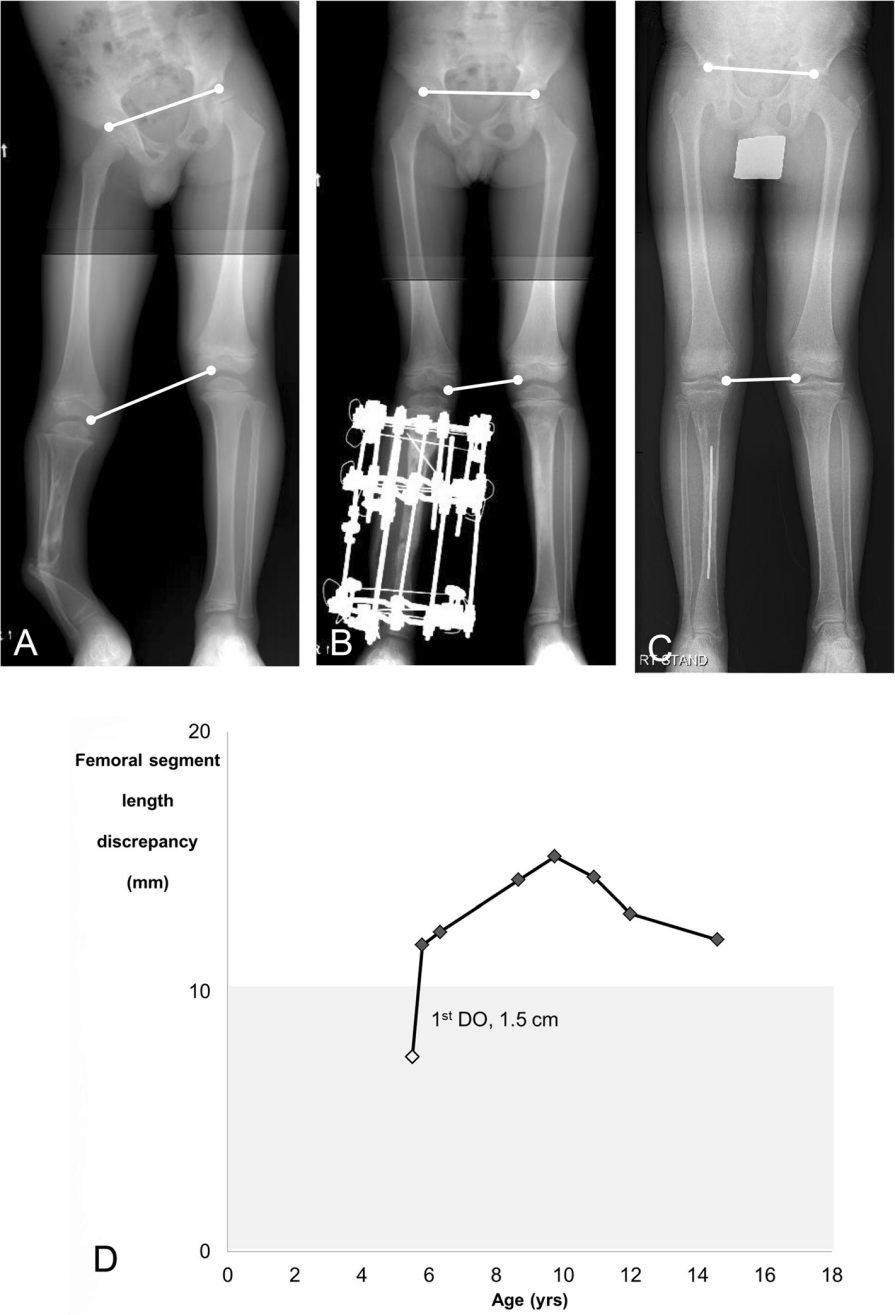
An example of Type B femoral overgrowth in a 14.6-year-old boy (Patient 13). **a** No femoral overgrowth of the affected limb was observed at age 5.5 years. **b** Femoral overgrowth of the affected limb was initiated during distraction osteogenesis. **c** Femoral overgrowth persisted until preadolescence. **d** The pattern of femoral overgrowth was classified as the upward slope-deceleration pattern (modified Shapiro Type 2) [16]. The white diamond indicates the point at which the patient underwent distraction osteogenesis

**Fig. 2:**
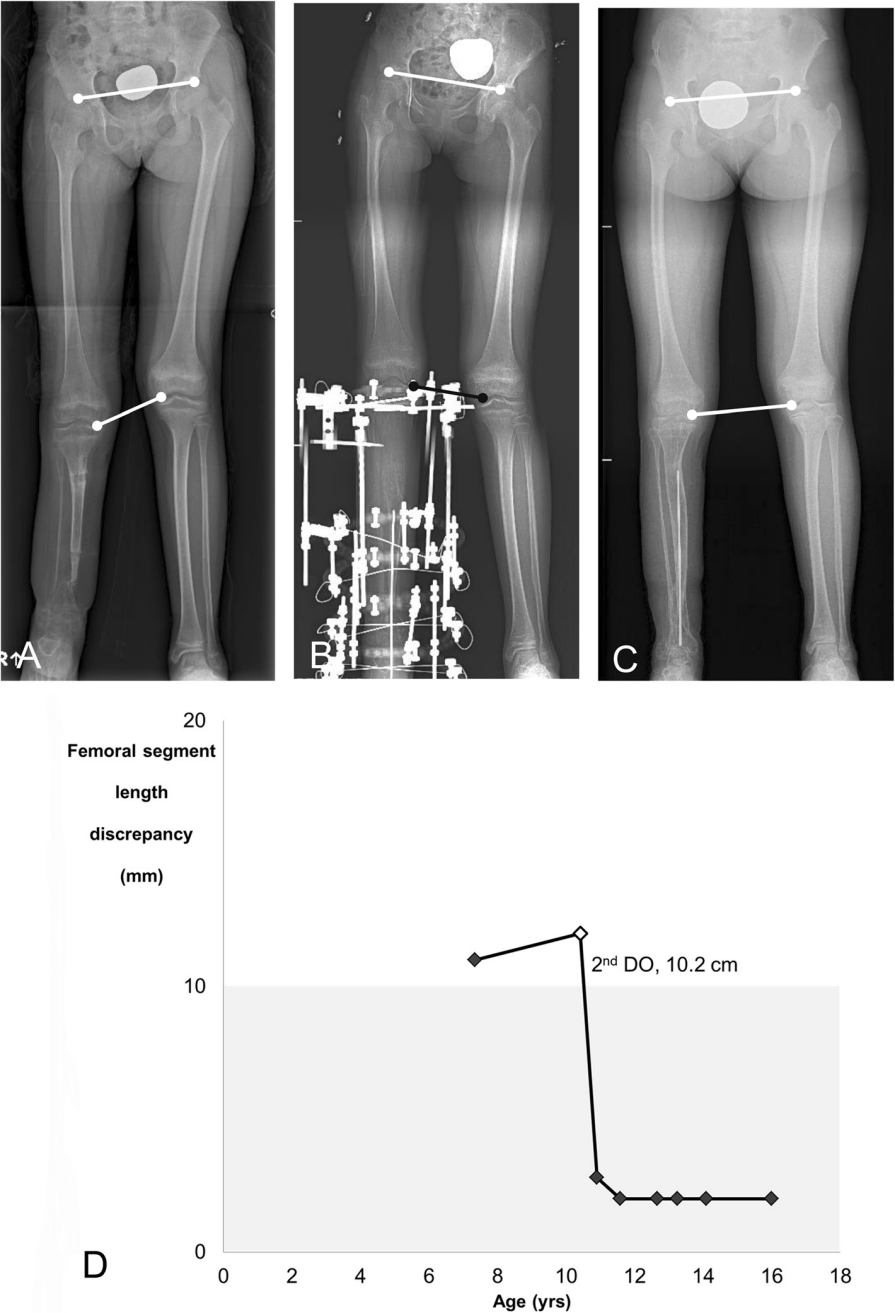
An example of Type C femoral overgrowth in a 16-year-old girl (Patient 16). **a** At age seven years, the patient, exhibiting 14 mm of femoral overgrowth of the affected limb, had failed to achieve union of the tibia via bone transport using the Ilizarov method and was referred to our institution. **b** After the patient underwent distraction osteogenesis, the femoral overgrowth resolved. **c** No significant femoral overgrowth was evident after the patient reached skeletal maturity. **d** The pattern of femoral overgrowth did not correlate with any of the subtypes defined by Shapiro’s classification [16]. The white diamond indicates the point at which the patient underwent distraction osteogenesis

At initial presentation, 11 patients exhibited a mean of 13 mm (10–23) of FO. Tibial shortening was 32 mm (−6–77), and shortening in foot height was 6 mm (6–8). However, the nature of FO changed over time for certain patients. At the last follow-up, 14 patients presented with FO; the average FO was 12 mm (10–18), and the affected tibia was 9 mm shorter (−18–48) than the contralateral tibia. Overall, 22 patients exhibited ≥10 mm of FO at least once during the follow-up period. Six cases were classified as Type A FO; seven as Type B (Fig. 1); four as Type C (Fig. 2); and four as Type D. Table 2 summarizes information regarding the 22 patients with FO at least once.

**Table 2:** Summary of 22 children who demonstrated femoral overgrowth (FO) of the affected limb during follow-up

Patient	Sex	At initial presentation			Number of DO treatments	Amount of DO (mm)	At last follow-up			Nature of FO^c^	Modified Shapiro’s Type
		Age (years)	Segment length discrepancy (mm)				Age (years)	Segment length discrepancy (mm)			
			Femur (Length-gain effect)^b^	Tibia				Femur (Length-gain effect)^b^	Tibia		
1^a^	F	7.3	+10 (0)	−9	none	0	16.3	+10 (0)	−6	A	3
2^a^	F	1.3	+17 (3)	−48	1	16	15.0	+17 (3)	−4	A	3
3^a^	F	0.8	+12 (4)	−61	1	52	7.7	+10 (3)	−13	A	3
4^a^	M	0.8	+10 (6)	+2	1	30	10.0	+10 (4)	−3	A	3
5^a^	M	1.0	+10 (3)	−6	none	0	17.0	+13 (3)	−3	A	4
6^a^	M	5.0	+15 (3)	−2	1	15	17.3	+10 (3)	−22	A	unclassifiable
7^a^	M	3.2	+10 (4)	−3	1	12	12.0	+10 (2)	−13	A	3
8^a^	M	1.0	+7 (3)	−30	3	90	16.0	+13 (3)	−48	B	4
9^a^	F	0.1	+5 (4)	−98	1	53	13.9	+11 (1)	−28	B	2
10^a^	M	6.1	0 (2)	−6	1	50	20.4	+10 (2)	−3	B	1
11^a^	F	0.8	0 (3)	−13	1	35	19.1	+12 (3)	−19	B	4
12^a^	M	5.3	+6 (4)	−10	1	17	17.3	+18 (6)	+1	B	2
13^a^	M	5.5	+8 (5)	−35	1	15	14.6	+12 (3)	−18	B	2
14^a^	F	6.6	+3 (4)	−18	1	15	17.6	+15 (3)	−28	B	3
15^a^	F	7.1	+23 (3)	−48	3	111	16.3	0 (2)	−25	C	unclassifiable
16^a^	F	7.0	+14 (3)	−75	2	102	16.0	+5 (4)	−21	C	unclassifiable
17^a^	M	1.0	+11 (4)	−59	2	76	8.3	+4 (4)	+4	C	unclassifiable
18^a^	F	0.2	+10 (1)	−41	1	33	7.5	0 (1)	+6	C	unclassifiable
19^a^	M	1.3	+5 (2)	−10	1	69	16.0	+7 (4)	−3	D	5
20^a^	F	2.9	+3 (4)	−90	1	38	16.1	+8 (4)	−19	D	5
21^a^	F	3.7	0 (1)	−46	1	15	14.1	+2 (2)	−21	D	5
22^a^	F	0.3	+2 (1)	−10	none	0	13.8	+5 (0)	−12	D	5

DO, distraction osteogenesis

Data represent the discrepancy of the femur and tibia (+, longer in the affected limb; −, shorter in the affected limb)

^a^Patient 1 was the only patient with prepseudarthrosis; all of the other patients presented with atrophic-type CPT

^b^Length-gain effect was defined as the effect of an increased femoral neck-shaft angle on FO. The length-gain effect was calculated by subtracting the distance between the summit of the femoral head and the mid-level of the lesser trochanter of the unaffected limb from the distance between the summit of the femoral head and the mid-level of the lesser trochanter of the affected limb

^c^The nature of FO was classified as follows: FO that was consistent from the initial presentation to the last follow-up (Type A); FO that was not observed at the initial presentation but that developed during treatment and remained consistent until the last follow-up (Type B); FO that was observed at the initial presentation but that was not apparent during follow-up (Type C); and FO that developed after the initial presentation and subsequently resolved (Type D)

### Contributors associated with femoral overgrowth

Only development of frank pseudarthrosis, extent of tibial shortening, DO treatment, and increased femoral neck-shaft angle were included in multivariate logistic regression analysis because the *p*-values of each of these parameters were <0.05 on the univariate analysis (*p* = 0.004, *p* = 0.046, *p* < 0.001, and *p* = 0.002, respectively). Frank pseudarthrosis, DO treatment, and increased femoral neck-shaft angle were significantly associated with FO (*p* = 0.016, *p* = 0.001, and *p* = 0.005, respectively).

The development of frank pseudarthrosis significantly contributed to FO (odds ratio [OR], 13.650; 95 % confidence interval [CI], 1.632 to 114.191; *p* = 0.016). Notably, the incidence of FO was significantly higher in patients with atrophic-type CPT than in patients with prepseudarthrosis; 13 (39.4 %) patients with atrophic-type CPT exhibited FO at the last follow-up, whereas only one (4.5 %) patient with prepseudarthrosis exhibited FO (Table 1).

DO treatment was another significant contributor to FO (OR, 14.500; 95 % CI, 2.809 to 74.837; *p* = 0.001). The per-patient numbers of DO treatments are presented in Table 2. Patients with Types B and D FO commonly underwent only one round of DO: 9 patients underwent one round of DO, and 1 patient underwent three rounds. However, most of the patients with Type C FO underwent more than two rounds of DO: 1 patient underwent three rounds of DO, 2 patients underwent two rounds, and 1 patient underwent one round.

An increased femoral neck-shaft angle of the affected limb was also significantly associated with FO (OR, 7.897; 95 % CI, 1.878 to 33.203; *p* = 0.005). The effect of an increased femoral neck-shaft angle on FO (length gain effect) is presented in Table 2 (range, 0–6 mm). When present, increased femoral neck-shaft angle of the affected limb exhibited a consistent trend from the initial presentation (mean, 15°; range, 10–31°) to the last follow-up (mean, 11°; range, 10–24°) and was not affected by the extent of tibial/fibular length gain.

### Patterns of femoral overgrowth

Patterns of FO were also evaluated for the 22 patients who exhibited ≥10 mm of FO at least once during the follow-up period (Table 2). Only one femoral segment displayed an upward linear slope growth-stimulated pattern (modified Shapiro Type 1); three femoral segments had upward slope-deceleration patterns; six femoral segments had upward slope-plateau patterns; three femoral segments had upward slope-plateau-upward slope patterns; and four femoral segments had upward slope-plateau-downward slope patterns. We were unable to correlate the patterns of five femoral segments with Shapiro’s developmental patterns [16].

## Discussion

To the best of our knowledge, this is the first report to systematically investigate the incidence of, contributors to, and patterns of FO of the affected limb in CPT. Our study demonstrates that FO is not infrequently observed in patients with atrophic-type CPT.

Information about FO of the affected limb in CPT is rare in the literature. Only two previously published reports briefly mentioned FO in CPT [5, 8]. Iamaguchi et al. observed an average of 20 mm (5–50) of FO in 25 % (4/16) of Brazilian patients with atrophic-type CPT [8]. Horn et al. reported that only a single Norwegian patient with prepseudarthrosis had 11 mm of FO; however, 52 % (10/19) of Norwegian patients with atrophic-type CPT exhibited a mean of 20 mm (14–29) of FO [5]. In comparison, our study found that 4.5 % (1/22) of patients with prepseudarthrosis presented 10 mm of FO during 9 years of longitudinal follow-up, whereas 39.4 % (13/33) of patients with atrophic-type CPT exhibited an average of 12 mm (10–18) of FO. Based on a compilation of all of the data from previous reports and our own study, it is evident that atrophic-type CPT demonstrates a significantly higher incidence of FO compared with prepseudarthrosis.

In previous reports, Iamaguchi et al. [8] and Horn et al. [5] postulated that the mechanism of FO reflects a compensatory response. We agree with these authors’ opinion that FO is likely a compensatory response to marked tibial shortening in patients with atrophic-type CPT. However, compensation alone cannot explain the reason(s) why only a fraction of such patients demonstrate FO at initial presentation or during treatment. Given that longitudinal long bone growth is affected by complex mechanisms with multiple factors, e.g., mechanical, hormonal, electrochemical, nutritional, genetic, and other factors [2], the FO observed in patients with CPT may be a reaction to those multiple influences. Our study clearly demonstrates that DO treatment coupled with osteosynthesis for atrophic-type CPT could influence the development of FO. This finding supports the notion that hyperemic stimulation after tibial osteotomy may induce FO of the affected limb, given that regional blood flow increases 10-fold during the distraction period and doubles until 17 weeks after the operation [17, 18]; moreover, blood flow also increases at sites distant from the osteotomy [17]. In fact, 10 of the 11 patients (90.9 %) who initially presented without FO but who later developed it (Type B and Type D patients) had undergone DO; most of these patients (9/10) underwent only one round of DO. Seven of these 10 patients exhibited FO initiated during the tibial lengthening process. However, DO did not always stimulate FO. In four patients, the initial FO was no longer apparent during follow-up (Type C). All but one of these patients underwent several rounds of DO and regained >35 % of the tibial length after distraction. We inferred that repeated and extensive limb lengthening may induce growth disturbance in these patients, a conclusion that was concurrent with a previous report on lengthening in children with achondroplasia [19]. Compressive forces across the physis or damage to the blood supply around the physis have been proposed as the cause(s) of the growth disturbance [19]. We suggest that undergoing tibial lengthening only once may stimulate FO, whereas repeated and extensive lengthening might inhibit FO.

Neurofibromatosis appears to be another contributor to FO in CPT. In this study, an increased femoral neck-shaft angle of the affected limb was significantly associated with FO. In addition, the tendency towards increased femoral neck-shaft angle, with a resultant length-gain effect, persisted throughout follow-up, regardless of the extent of the tibial length gain. Despite the absence of a direct association between the coexistence of NF1 and FO, we postulated that the increased femoral neck-shaft angle of the affected limb might have originated from NF1 because overgrowth phenomena and coxa valga are clinical characteristics of neurofibromatosis [1, 2, 16]. In fact, only one patient, who exhibited FO in the prepseudarthrotic stage, concurrently had NF1.

We found that the patterns of FO in our series were quite different from those previously reported by Shapiro [16] in neurofibromatosis, which is closely related to CPT. He reported that the limb length discrepancy in most children with neurofibromatosis showed a linear pattern of progression. However, the upward slope-plateau pattern (modified Shapiro Type 3) was most common in our study population, and only one patient showed an upward linear slope growth-stimulated pattern (modified Shapiro Type 1). The upward slope-deceleration pattern, the upward slope-plateau-upward slope pattern, and the upward slope-plateau-downward slope pattern were also observed. Five cases could not be classified according to Shapiro’s developmental patterns. Diverse patterns of FO in our series may have resulted from complex interactions involving a compensatory response to frank pseudarthrosis, DO treatment, and neurofibromatosis.

This study has several limitations. First, the measurement of actual segment length for a tibia with anterolateral bowing is impractical, especially in patients with atrophic-type CPT; hence, in this study, we measured effective tibial length [11] instead of actual tibial length. However, the effective tibial length may be affected by the amount of the loaded weight; we inferred that this is the reason why the extent of tibial shortening did not correlate with FO in the current study. In addition, the method for measuring the femoral neck-shaft angle may have been influenced by femoral anteversion, which was not precisely addressed. Therefore, to improve statistical significance, we defined an increase of ≥10° in the femoral neck-shaft angle of the affected limb to be an increased femoral neck-shaft angle. Finally, in our series, no specific case exhibited FO initiation when frank pseudarthrosis was established, although the development of frank pseudarthrosis noticeably contributed to FO in the multivariate analysis. These limitations were inevitable because of the retrospective study design. In future studies, a prospective approach should be considered.

## Conclusions

Our observations indicate that FO of the affected limb is frequently encountered in patients with atrophic-type CPT. A compensatory response to frank pseudarthrosis, DO treatment, and neurofibromatosis may play a role in the diverse patterns of FO.

## Abbreviations

AP, anteroposterior; CPT, congenital pseudarthrosis of the tibia; DO, distraction osteogenesis; FO, femoral overgrowth; NF1, neurofibromatosis type 1.
